# Effectiveness and Safety of Over-the-Counter Tooth-Whitening Agents Compared to Hydrogen Peroxide In Vitro

**DOI:** 10.3390/ijms24031956

**Published:** 2023-01-19

**Authors:** Lena Katharina Müller-Heupt, Nadine Wiesmann-Imilowski, Sebahat Kaya, Sven Schumann, Maximilian Steiger, Monika Bjelopavlovic, James Deschner, Bilal Al-Nawas, Karl Martin Lehmann

**Affiliations:** 1 Department of Oral and Maxillofacial Surgery, University Medical Center Mainz, Augustusplatz 2, 55131 Mainz, Germany; 2 Department of Otorhinolaryngology, University Medical Center Mainz, Langenbeckstr. 1, 55131 Mainz, Germany; 3 Institute of Anatomy, University Medical Center Mainz, Langenbeckstr. 1, 55131 Mainz, Germany; 4 Department of Prosthetic Dentistry, University Medical Center Mainz, Augustusplatz 2, 55131 Mainz, Germany; 5 Department of Periodontology and Operative Dentistry, University Medical Center Mainz, Augustusplatz 2, 55131 Mainz, Germany

**Keywords:** bromelain, enzymatic whitening, hydrogen peroxide, sodium bicarbonate, sodium chlorite, teeth-whitening, PAP, phthalimidoperoxycaproic acid

## Abstract

(1) This study investigated the whitening effect, cytotoxicity and enamel surface alterations induced by different over-the-counter (OTC) bleaching agents in comparison to hydrogen peroxide. (2) Human teeth (n = 60) were randomly assigned into 6 groups (n = 10), stained with coffee solution for 7 d, followed by a whitening period of 7 d with either placebo, bromelain, sodium bicarbonate, sodium chlorite, PAP or hydrogen peroxide. Color measurements were performed with a spectrophotometer. Scanning electron micrographs (SEM) were taken to assess the enamel structure. Cytotoxicity of the tested substances was assessed based on the cell viability of primary human fibroblasts. (3) The application of all whitening gels resulted in a greater color difference of the enamel (ΔE) in comparison to the negative control. Hydrogen peroxide caused the greatest color difference. Bromelain and PAP treatment showed no enamel surface changes, in contrast to hydrogen peroxide treatment, which showed very mild interprismatic dissolution. Bromelain was the only non-cytotoxic agent. (4) The maximum effect achieved by all OTC bleaching agents was the removal of stains, whereas hydrogen peroxide was capable of further whitening the teeth. Bromelain treatment was neither cytotoxic, nor resulted in enamel surface alterations, and its whitening effect was less, yet still effective, compared to hydrogen peroxide.

## 1. Introduction

Tooth-whitening has become a highly sought-after in-office dental treatment [1]. Beyond that, the market for over-the-counter (OTC) whitening systems has increased over the last years [2], due to a rising demand for easy and home-based aesthetic procedures. Dental in-office bleaching systems are primarily based on hydrogen peroxide (H_2_O_2_) or one of its precursors, such as carbamide peroxide (CP). CP is used in different concentrations in both products for in-office treatment and home bleaching. It has a slower degradation rate compared to H_2_O_2_ and, since it is usually applied to the tooth surface via dental trays, it is in contact with the surrounding tissue for a longer period of time compared to H_2_O_2_ [3]. Peroxide-based products used for in-office bleaching are applied directly to the enamel surface in combination with a cheek retractor and protection of the soft tissue. Peroxides release highly reactive free radicals, leading to oxidization of organic chromophores, small molecules from coffee, red wine or tea [4]. Thus, these are broken down into smaller molecules and absorb fewer wavelengths of visible light, resulting in a lighter appearance of the teeth [5].

Tooth-whitening with peroxides is generally considered a safe and effective procedure. However, it is known to temporarily increase teeth sensitivity, which affects 43% to 80% of patients after whitening their teeth with peroxides [6,7,8]. This effect is most likely due to microscopic surface damage of the enamel, allowing oxygen radicals to diffuse towards the dental nerve and damage cells [8,9,10], which leads to temporary dental nerve inflammation [11]. Nevertheless, it remains unclear whether the tooth enamel is damaged by bleaching procedures with peroxides. Some studies report changes in the enamel surface caused by bleaching with peroxides [12,13,14,15,16,17,18,19,20], while others did not report any surface alterations [14,15,18,21]. In vitro studies have demonstrated demineralization effects, such as a reduction of the mineral content or changes in the calcium/phosphate ratio [22,23,24,25,26]. The mineral loss was found down to a depth of 250 μm below the enamel [25,26].

Within the European Union, products containing > 0.1% peroxides are not allowed for individual sale and their in-office use and prescription with individualized trays is limited to dentists. Therefore, OTC teeth-whitening products on the market contain a range of other active ingredients, such as phthalimidoperoxycaproic acid (PAP), sodium chlorite or sodium bicarbonate. PAP, an organic peroxide, oxidizes chromogens without the formation of free radicals, due to epoxidation of molecules containing conjugated double bonds [27]. Sodium chlorite decomposes into chlorine dioxide and the active bleaching agent in the presence of acid [28]. Sodium bicarbonate is mainly known as an abrasive substance in whitening toothpastes [29], but it is also sold as an active ingredient in OTC whitening gels. According to the instructions for use, sodium bicarbonate contained in whitening gels should be applied onto the tooth surface and thus do not exert an abrasive effect. Clinical studies have shown that dentifrices containing sodium bicarbonate were more effective regarding teeth-whitening, compared to even more abrasive substances [30,31,32]. For this reason, there may be an additional chemical whitening mechanism of sodium bicarbonate which is not yet fully understood.

In general, there is low evidence regarding the effectiveness and safety of OTC teeth-whitening agents [2,33]. A recent meta-analysis compared several laboratory studies and demonstrated that non-peroxide bleaching agents were less effective compared to peroxide-based bleaching agents [34]. Some sodium chlorite and PAP formulations have been shown to reduce enamel microhardness and alter the enamel surface in vitro [33], hypothetically due to their low pH level. Therefore, in another in vitro study, the PAP formulation was changed, its pH was adjusted to 6.5–7.0 and hydroxyapatite was included. In this study, no reduction of enamel microhardness was observed for the adjusted PAP formulation [27]. 

In the recent literature, natural compounds, such as enzymes, have been listed as alternative whitening agents. Bromelain, papain and cysteine proteases of natural origin have recently been tested as whitening gels in vitro and as dentifrices in vivo and in vitro [35,36,37,38]. Cysteine proteases chemically break down proteins by cleaving peptide bonds and thus may represent a possible alternative for whitening teeth [4]. The cleavage of peptide bonds changes the light reflection [34], which leads to a lighter appearance. Thus, the application of bromelain seems to be an interesting approach for OTC teeth-whitening and differs from existing whitening ingredients, since the primary mechanism of action is not an oxidation. 

Regarding OTC whitening products, there is a lack of consistent research protocols and data regarding their safety and efficacy. This is of special interest, since OTC whitening products and active ingredients sales, both in the EU and globally, are increasing. Thus, the present study assessed the efficacy of natural and non-natural non-peroxide whitening agents in comparison to hydrogen peroxide on human teeth. 

This study was designed to assess the efficacy and safety of different OTC whitening agents in comparison to hydrogen peroxide. 

## 2. Results

### 2.1. Color Changes

**Figure 1:**
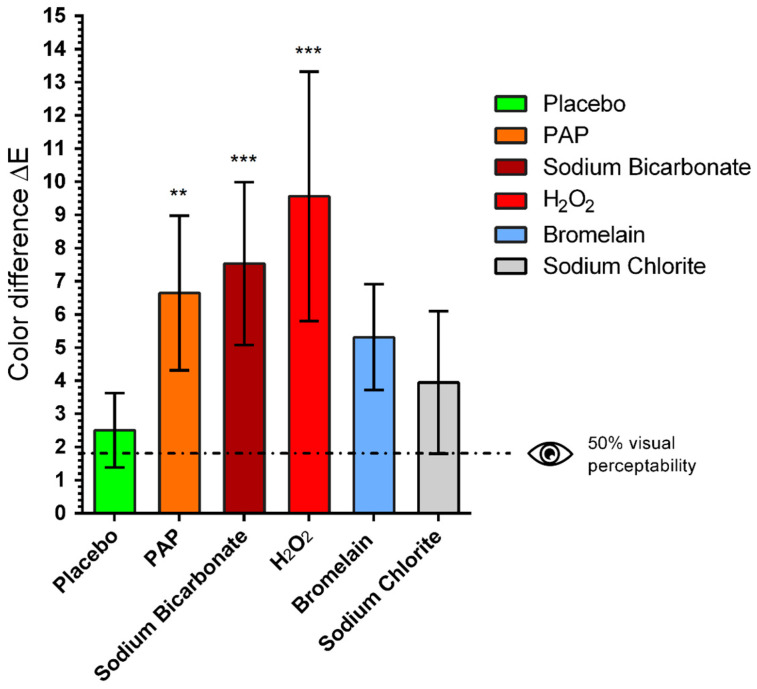
Mean values and standard deviations of the color difference (ΔE) after whitening procedure with different gels: 12% phthalimidoperoxycaproic acid (PAP), 5% sodium bicarbonate (Sodium Bicarbonate), 6% hydrogen peroxide (H_2_O_2_), 1% bromelain (Bromelain), 0.2% sodium chlorite (Sodium Chlorite). The value indicated by the dotted line at ΔE = 1.8 corresponds to 50% visual perceptibility under clinical conditions and was used here as a reference for visually detectable color changes. One-way ANOVA, comparison of each treatment group with untreated control group, ** *p* < 0.01, *** *p* < 0.001.

The application of all whitening gels resulted in a greater color difference (ΔE) than the negative control (placebo). H_2_O_2_ produced the strongest color change (ΔE = 9.6, *** *p* < 0.001), followed by sodium bicarbonate (ΔE = 7.5, *** *p* < 0.001) and PAP (ΔE = 6.6, ** *p* < 0.01), bromelain (ΔE = 5.3, not significant) and sodium chlorite (ΔE = 3.9, not significant), as shown in Figure 1. The value indicated by the dotted line at ΔE = 1.8 corresponds to 50% visual perceptibility under clinical conditions and was used as a reference for visually detectable color changes.

The color difference (ΔE) was calculated according to the CIELab system [39]. For all bleaching agents, ΔL and Δb were both reduced, whereas for bromelain mainly Δb was reduced (Table 1).

### 2.2. Microscopic Inspection by SEM 

**Figure 2:**
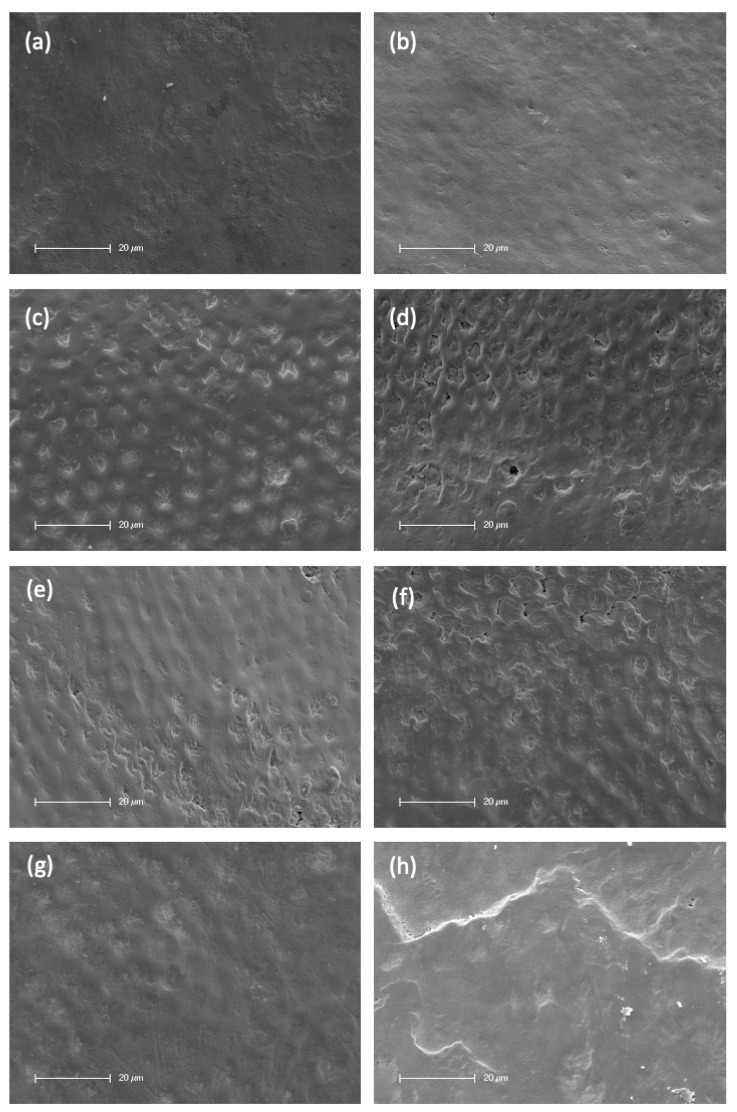
SEM images of control enamel (**a**,**c**,**e**,**g**) compared to the other side of the same tooth after treatment ((**b**) = Bromelain, (**d**) = H_2_O_2_, (**f**) = PAP, (**h**) = Sodium Bicarbonate). Magnification 1200×. Scale 20 µm. N = 2 per treatment.

To compare the surface morphology of the different specimens, scanning electron micrographs (SEM) were performed for both control and treatment groups. Sound enamel showed the typical keyhole configured hydroxyapatite crystallites as seen in Figure 2a,c,e,g. Teeth that were treated with bromelain and PAP showed no enamel surface changes (Figure 2b,f), while H_2_O_2_ treatment resulted in very mild interprismatic dissolution (Figure 2d) in comparison to the untreated side of the same tooth (Figure 2c). Teeth treated with sodium bicarbonate (Figure 2h) showed a flat layer with granules on their enamel surface in comparison to the control enamel (Figure 2g).

### 2.3. Cellular Viability 

**Figure 3:**
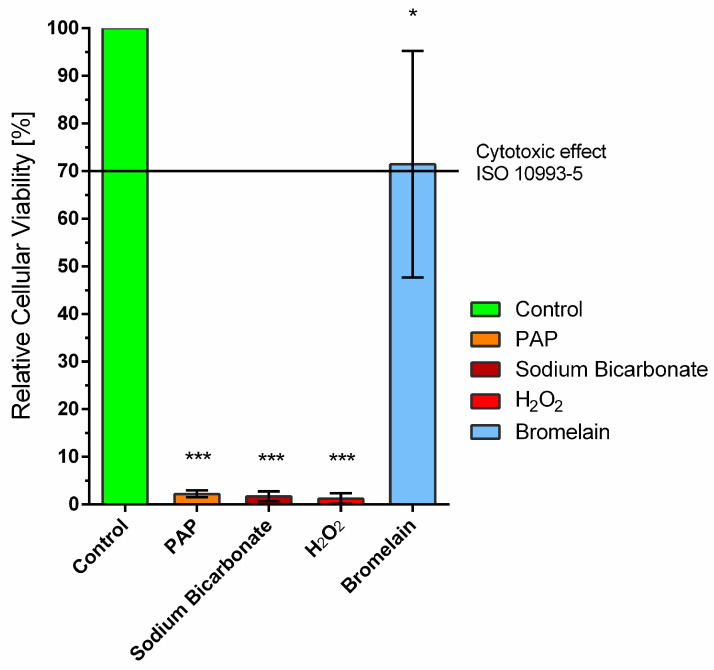
Cellular viability of primary human fibroblasts treated with different whitening agents. These were 12% phthalimidoperoxycaproic acid (PAP), 5% sodium bicarbonate (Sodium Bicarbonate), 6% hydrogen peroxide (H_2_O_2_), 1% bromelain (Bromelain). Oral primary human fibroblasts in a pure culture medium which were not exposed to eluates served as control. One-way ANOVA, comparison of each treatment group with untreated control group * *p* < 0.05, *** *p* < 0.001.

The relative cellular viability of primary human fibroblasts incubated with different whitening agents is shown in Figure 3. Oral primary human fibroblasts in pure culture medium which were not exposed to any eluates served as control, and their metabolic activity was considered 100% cell viability. PAP, sodium bicarbonate and H_2_O_2_ exerted a cytotoxic effect against fibroblasts (*p* < 0.001). Bromelain was the only non-cytotoxic bleaching gel and presented a cell viability of >70%, which is defined as non-cytotoxic by ISO 10993-5.

## 3. Discussion

This study was designed to assess the efficacy and safety of different OTC whitening agents in comparison to hydrogen peroxide. Our study revealed that H_2_O_2_ was the most effective agent for teeth-whitening (ΔE = 9.6). These findings are consistent with the results of a meta-analysis, which concluded that peroxide-based bleaching were more effective than non-peroxide bleaching agents [34]. Furthermore, it was the only agent capable of not only removing artificial stains, but further whitening the natural enamel color. However, enamel treated with H_2_O_2_ showed mild interprismatic dissolution. The maximum whitening effect achieved by the OTC whitening agents was the removal of artificially induced stains. Both PAP and bromelain reduced artificially induced enamel stains and did not cause any surface alterations. Bromelain was found to be the only non-cytotoxic whitening agent.

Regarding teeth-whitening, H_2_O_2_ was the most effective agent (ΔE = 9.6, *p* < 0.001), followed by sodium bicarbonate (ΔE = 7.6, *p* < 0.001) and PAP (ΔE = 6.6, *p* < 0.01). Furthermore, H_2_O_2_ was the only agent capable of not only removing artificial stains, but further whitening the natural enamel color detected before the staining procedure. Peroxides react with organic materials within the tooth structure [5], and their whitening mechanism may be more effective than those obtained by OTC whitening agents. Regarding enamel surface alterations, H_2_O_2_ showed mild interprismatic dissolution compared to sound enamel, whereas bromelain and PAP treatment resulted in no surface alterations, as shown by the scanning electron micrographs. Sodium bicarbonate appeared to adhere to the enamel surface. A flat layer with granules on the enamel surface was observed in comparison to the control sound enamel.

H_2_O_2_ has a low molecular weight (34 g/mol) and has the capability to penetrate into enamel [40]. Enamel surface alterations induced by H_2_O_2_ have been reported in many studies [12,13,14,15,16,17,18,19,20], but most of these changes have not been seen in studies where teeth were stored in saliva or remineralizing agents [41,42,43]. In our study, mild interprismatic dissolution was observed, even though teeth were stored in artificial saliva. To minimize structural enamel differences between teeth and individuals, two samples from two surfaces of each tooth—treated and untreated—were chosen. 

The maximum effect achieved by all OTC whitening agents was the removal of stains. In contrast, Pascolutti et al. observed higher color changes for PAP than for 6% hydrogen peroxide [27]. In their study, a complex of different polyphenols, including tea, red wine and ferric chloride was used for the staining procedure, and the authors hypothesized that those stains may be more difficult to decolorize by free radicals, due to their antioxidant activity. Furthermore, enamel surfaces were etched with 1% HCl to remove external stains prior to the staining period, whereas, in our study, teeth were polished. The etching process may have influenced the enamel surface and, thus, changed the susceptibility to whitening gels.

In our study, changes in ΔL* (lightness/brightness) and Δb* (yellow hue) were observed for all whitening agents. The whitening effect of bromelain (ΔE = 5.3) was not significant and observed changes in ΔL* were minimal, whereas larger differences in Δb* were observed. In contrast, in a study by Münchow et al., bromelain-similar reductions were observed in ΔL* and Δb* [37]. This may be explained by either a different application protocol (once per week, three times per day for four weeks) or by the usage of bromelain obtained from soy proteases, whereas, in our study, bromelain from the pineapple stem was used. In another study, bromelain almost had the same whitening efficacy as CP [38]. The pH of the gel was adjusted to 7.2–7.5, the optimum pH for proteolytic activity of the enzyme bromelain in this study, whereas, in our study, the pH of the gel was 6.5, since the optimum pH range for the enzyme used in this study was 6.0–6.8, according to the manufacturer. This may have resulted in a different enzymatic activity and thus, lower whitening efficacy of the bromelain. Furthermore, in our study, human teeth instead of bovine teeth were used, which may contribute to slightly different results. No cytotoxicity to mouse fibroblasts [38] and no damage to enamel [38] were observed in bromelain gel formulations previously used in the literature, which is consistent with our results in this study. Therefore, bromelain may be a safe, yet effective, ingredient in OTC oral care products for enzymatic stain removal. Nevertheless, the potential of allergic reactions of bromelain should be discussed, since it can easily be swallowed if used in oral care products. Airway sensitizations with immunological mechanisms, such as the detection of specific IgE antibodies, have been reported for industrial workers exposed to bromelain dusts [44]. Furthermore, bromelains may cause immediate- or late-type reactions with predominantly respiratory symptoms and are known to be a strong sensitizer, but sensitization occurs due to inhalation and not to ingestion [45].

Enzymatic whitening agents, alone or as additive, may be an interesting approach as OTC whitening agent alone or in combination with peroxides. A study by Vekaash et al. has shown a higher bleaching efficacy if peroxides were combined with pineapple extract [46]. The combination may not only enhance the bleaching efficacy of other whitening agents, but furthermore reduce their unwanted side effects. Further studies are necessary to clarify the exact molecular whitening mechanisms to find optimal combinations and to extend the enzymatic activity and stability of natural bromelain preparations.

Sodium bicarbonate resulted in effective teeth-whitening of ΔE = 7.6 (*p* < 0.001) in our study. It is commonly used in dentifrices, due to its abrasive properties, which leads to removal of extrinsic stains [32]. In our experimental setting, the gel was not brushed and, thus, the whitening effect of sodium bicarbonate observed cannot be attributed to its abrasivity. A whitening effect, additionally to its abrasive properties, was observed in a clinical study comparing silica-based dentifrices with bicarbonate-based dentifrices by Koertge et al. [47]. Regardless, the exact whitening mechanisms of sodium bicarbonate, apart from its abrasive effect, remain unknown. Dental enamel comprises approximately 2–4% carbonate [48], and spectroscopic analyses indicated that carbonate ions from bicarbonate can be incorporated into surface layers [49]. Therefore, its whitening effect—aside from any abrasive effects—may be attributed to its ability to adhere to the enamel surface, which is in line with our observations in the SEM micrographs. This effect may be similar to the effect of hydroxyapatite, which also has the ability to adhere to enamel and, thus, increase ΔE [50]. Nevertheless, the whitening effect of sodium bicarbonate should be subject to further studies to clarify its exact whitening mechanism. 

Furthermore, we observed that enamel of different subjects reacted differently towards the staining procedure, even though it was standardized regarding time, coffee solution and initial polish. The relative color change of the teeth seemed to be individual and could not be related to their initial color. Individual efficacy of teeth-whitening may therefore be potentially attributed to the molecular structure of the enamel. In this regard, the permeability of dental tissue seems to play an important role in the process of color changes [51]. However, there is a lack of evidence regarding the influence of individual factors of enamel microstructure on the staining or bleaching procedures. In our study, there may have been individual differences on the molecular structure of the enamel, such as the patients age, microscopic surface erosions and the location of the extracted tooth. Nevertheless, no relation between staining and bleaching could be observed, since enamel that stained very easy did not necessarily whiten again easier. Further studies on enamel microstructure and susceptibility for staining and whitening should be performed. 

## 4. Materials and Methods

### 4.1. Specimen Preparation

A total of 60 human teeth (incisors, canine teeth, premolars, molars) were extracted for therapeutic reasons, and obtained from patients who underwent surgery at the Department of Maxillofacial and Plastic Surgery, University Medical Center Mainz, Germany. This study was performed in agreement with the declaration of Helsinki on the use of human material for research. In accordance with the ethics committee of Rhineland-Palatinate, patients agreed with the scientific use of the surplus material and no further approval of the medical ethics committee was required, as the teeth were used anonymously. The teeth were visually inspected and were free of caries, cracks or restorations. Tissue remnants were manually removed using 70% ethanol (Roth, Karlsruhe, Germany). After cleaning with distilled water, the teeth were wet-polished with proxyt prophy-paste (RDA 36, ivoclar vivadent). After polishing, the enamel was etched using 36% phosphoric acid (Dentsply Sirona, Bensheim, Germany) for 60 s, and rinsed with distilled water for 30 s, in order to remove the smear layer and enhance staining into the enamel, according to a protocol by Meireles et al. [39]. Following the specimen preparation there were two phases of the experiment:

1. staining procedure
2. whitening procedure

Teeth were maintained in artificial saliva (apomix, Halle, Germany) throughout the study to maintain a moist environment.

### 4.2. Staining Procedure

After preparation, the fissures and roots were sealed with clear varnish (cosnova, Sulzbach, Germany) to protect the dentin and pulp chamber from excessive staining. The entire enamel surface was left unvarnished. The teeth were stored in coffee (Senseo, Jacobs Douwe Egberts, Bremen, Germany) in an incubator at 37 °C (Heracell 240i, Thermo Fisher Scientific, Waltham, MA, USA) for 7 d. The coffee was freshly brewed and replaced everyday using 7 g of coffee and 200 mL of boiling distilled water pumped with 1 bar through the filter (Senseo, Jacobs Douwe Egberts, Bremen, Germany). 

### 4.3. Preparation of Bromelain Whitening Gel

A homogenous gel containing 1% wt bromelain (600,000 U/g, Selco, Wald-Michelbach, Germany) extracted from pineapple stems was prepared as described by Ribeiro et al. [38]. The formulation was prepared at room temperature. A homogenous gel was prepared using xanthan gum (200 mesh, v03trading, Willich, Germany) incorporated in propylene glycol (Dragonspice, Reutlingen, Germany) as thickener. Sodium fluoride (Chempur, Piekary Slaskie, Poland) and sodium benzoate (Chempur) were solubilized in ultrapure water. Bromelain was incorporated until a homogenous gel containing 1% wt was fabricated. The pH was adjusted to 6.5 by adding sodium hydroxide (AlginChemie, Neustadt-Glewe, Germany). The other gels, 5% sodium bicarbonate, 0.2% sodium chlorite, 12% PAP and 6% hydrogen peroxide, were purchased and stored according to the manufacturer’s instructions.

After 7 d of staining procedure, the teeth were randomly divided into the following five test groups (n = 10): 6% hydrogen peroxide (Zoom DayWhite, Philips, Amsterdam, The Netherlands), 0.2% sodium chlorite (Zhengzhou Huaer Electro-Optics Technology, Zhengzhou, China), 5% sodium bicarbonate (Nanchang Dental Bright Technology, Nanchang, China), 12% phthalimidoperoxycaproic acid (PAP, Nanchang Dental Bright Technology, Nanchang, China) and 1% bromelain. A negative control group (n = 10) was treated with a homogenous gel with distilled water and xanthan gum as thickener. The application protocol was chosen, according to previous studies with enzymatic-based whitening substances [37,38] and the recommended application time for OTC whitening gels. The gels were placed on the enamel surface, covered with a moistened gauze, and incubated for 16 min at 37°C. After 16 min, the gels were rinsed off with distilled water and specimens were maintained in artificial saliva. This procedure was repeated every 24 h for 7 d, simulating daily applications in a one-week treatment interval.

### 4.4. Spectrophotometric Color Determination

Color measurements were performed three times to obtain (I) the initial specimen color, (II) the color after 7 d staining procedure and (III) after 7 d whitening procedure. Measurements were performed with a spectrophotometer (Vita Easyshade, VITA Zahnfabrik GmbH & Co. KG, Bad Säckingen, Germany) and, for better comparability, always in the same area on the tooth surface. The L*, a* and b* color parameters were recorded and the differences between the tooth colors (i.e., color change, ΔE*) were calculated as ΔE* = $\sqrt{\left[{\left(\Delta L*\right)}^{2}+{\left(\Delta a*\right)}^{2}+{\left(\Delta b*\right)}^{2}\right]}$, where the a* value is defined as the red-green axis, the b* value as the blue-yellow axis and the L* value defines lightness. ΔL*, Δa* and Δb* corresponded to differences between the final and the baseline in the L*, a* and b* color parameters.

### 4.5. Microscopic Inspections

For microscopic inspections, teeth were cut in half using a diamond band saw (300 CL, Fa exakt, Norderstedt, Germany). Due to sputter coating, the same area could not be evaluated before and after treatment as described in the whitening procedure. To minimize bias effects to a minimum, one half of each tooth was treated according to the bleaching protocol, whereas the other half served as control. Two teeth per group were treated and inspected (n = 2). After sputter coating (Leica EM ACE200, Wetzlar, Germany) with gold in an argon atmosphere scanning electron microscope, visualization was performed with a Philips XL30 ESEM (environmental scanning electron microscope) system (Philips, Eindhoven, The Netherlands).

### 4.6. Cell Isolation and Cell Culture

Primary human fibroblasts were isolated from mucosa obtained from patients who underwent surgery at the Department of Otorhinolaryngology, University Medical Center Mainz, Germany. This study was performed in agreement with the declaration of Helsinki on the use of human material for research. In accordance with the ethics committee of Rhineland-Palatinate, patients agreed with the scientific use of the surplus material and no further approval of the medical ethics committee was required, as the fibroblasts were used anonymously. Tissue samples were cut into small pieces of approximately 2 × 2 mm with a sterile disposable scalpel. Prior to cell isolation, the tissue pieces were stepwise sterilized in 70% ethanol, in sterillium classic pure (Bode Chemie, Hamburg, Germany), and again in 70% ethanol. Then they were transferred to 5–10 mL (depending on the amount of tissue) 0.5% protease solution (P6141, Sigma-Aldrich, St. Louis, MO, USA) in phosphate buffered saline (PBS; Sigma-Aldrich, St. Louis, MO, USA) and incubated overnight at 4 °C. The next day, the protease solution was incubated with shaking for a further 15 min at 37 °C. The sample was then passed through a cell sieve (EASYstrainer 70 µm sterile, Greiner bio-one, Kremsmünster, Austria) with the help of a cell scraper (Falcon, Corning, NY, USA). Cells were pelleted by centrifugation (1500 rpm, 5 min), transferred to cell culture medium and seeded into small cell culture flasks with 25 cm^2^ grow area. Cells were characterized morphologically and were used, at most, until passage 10, to ensure primary identity. Cells were maintained in DMEM/Ham’s F12 (Gibco, ThermoFisher Scientific, Waltham, MA, USA) and supplemented with 10% fetal calf serum (PAA Laboratories, Pasching, Austria) and antibiotics (10,000 U/mL penicillin and 10 mg/mL streptomycin, Sigma-Aldrich, St. Louis, MO, USA) at 37 °C in 5% CO_2_.

### 4.7. Cellular Viability

Cells were seeded into a 96-well-plate (10,000 cells/well in 250 μL cell culture medium) and they were given time to adhere overnight. After 24 h, cells were treated with 200 µL of the different whitening gel solutions.

For this purpose, 1.5 mL DMEM/Ham’s F12 medium were mixed with 75 µL of each whitening gel in each well and placed in a 24-well-plate (Greiner Bio-One, Frickenhausen, Germany). After incubation for 45 min at 37 °C, as described in Ribeiro et al. [38], 200 µL of this medium was transferred to the 96-well-plates containing the cells. After 24 h of incubation, the cell medium was exchanged for medium with 10% AlamarBlue Cell Viability Reagent (ThermoFisher Scientific, MA, USA) and the cells were incubated for 4 h at 37 °C. Fluorescence was measured on a Fluorescence Microplate Reader (Fluoroskan Ascent Microplate reader, ThermoFisher Scientific, MA, USA). Results were given as relative fluorescence using a 538 nm excitation filter and a 600 nm emission filter, normalized to untreated control.

### 4.8. Statistical Analysis

Unless stated otherwise, results were expressed as mean +/− standard deviation (SD). The Kolmogorov–Smirnov normality test was used to determine if data sets were well-modelled by a normal distribution. In case of normal distribution, ANOVA test was used; otherwise, the Kruskal–Wallis test was used. A *p*-value of less than 0.05 was considered statistically significant. Correction for multiple comparisons was done by Bonferroni. Statistical significance is denoted as * *p* < 0.05, ** *p* < 0.01, *** *p* < 0.001. All statistical analyses were performed using Prism 6.0 for Windows (GraphPad Software, La Jolla, CA, USA). 

## 5. Conclusions

Our study revealed that H_2_O_2_ was the most effective whitening agent compared to bromelain, PAP, sodium bicarbonate and sodium chlorite. Nevertheless, mild surface alterations were observed on enamel treated with H_2_O_2_. Furthermore, H_2_O_2_ was the only whitening agent capable of not only removing artificial stains, but further whitening the natural enamel color. The maximum effect achieved by all OTC whitening agents used in this study was the removal of artificially produced stains. PAP and bromelain reduced artificially induced enamel stains and did not cause any surface alterations; thus, both substances may be used in OTC oral care products. Sodium bicarbonate was effective in reducing stains and seemed adherent to the enamel surface, which may explain its whitening mechanism, due to a modified light reflection of the tooth surface. Nevertheless, its whitening mechanism remains unclear and should be subject to further studies. 

Bromelain was the only non-cytotoxic whitening agent and, thus, may be a safe and effective ingredient in OTC oral care products for stain removal, even if used regularly and over a longer period, such as in toothpastes. Furthermore, our study showed individual susceptibility of the teeth regarding the amount of staining and whitening, which could not be correlated and may be attributed to the individual molecular enamel structure. This phenomenon should be subject to further studies, since it may play an important role in improving and individualizing teeth-whitening products according to a person’s individual enamel, to reduce side effects and maximize whitening efficacy. 

## Figures and Tables

**Table 1:** Color parameters L*, a* and b* of the different bleaching agents and calculated color differences (ΔE).

Whitening Agent	ΔL	Δa	Δb	ΔE
Placebo	1.4	0.3	0.0	2.3
PAP	4.1	0.9	4.9	6.6
Sodium Bicarbonate	4.6	1.4	4.5	7.5
H_2_O_2_	7.0	1.6	5.7	9.6
Bromelain	1.6	0.8	4.7	5.3
Sodium Chlorite	2.1	0.5	1.6	3.9

## Data Availability

Not Applicable.
